# Piezoelectric Condylectomy Through Transoral Endoscopic Approach: A Cadaveric Study

**DOI:** 10.1007/s12070-022-03168-0

**Published:** 2022-11-05

**Authors:** Giovanni Dell’Aversana Orabona, Vincenzo Abbate, Francesco Maffia, Antonio Romano, Paola Bonavolontà, Alessandra Valletta, Giorgio Iaconetta, Luigi Califano

**Affiliations:** 1 Maxillofacial Surgery Unit, Department of Neurosciences, Reproductive and Odontostomatological Sciences, University of Naples “Federico II”, Via Pansini 5, 80100 Naples, Italy; 2 Dentistry Unit, Department of Neurosciences, Reproductive and Odontostomatological Sciences, University of Naples “Federico II”, Via Pansini 5, 80100 Naples, Italy; 3 Neurosurgery Unit Department of Medicine, Surgery and Odontoiatrics, University of Salerno, Via Giovanni Paolo II 132, 84084 Fisciano Salerno, Italy

**Keywords:** Condylectomy, Piezosurgery, Transoral approach, Temporomandibular Joint, Minimally invasive surgery

## Abstract

Surgical approaches in the treatment of TMJ pathologies are a much-debated topic in literature. We propose a new surgical approach performed by intraoral access and completed by endoscopic magnification and long-tip piezosurgery assistance. A piezosurgery (Piezosurgery Plus, Mectron s.p.a. 2014) with a long angled tip (MT5-10 L) was used to perform an endoscopically assisted condylectomy.

**Supplementary Information:**

The online version contains supplementary material available at 10.1007/s12070-022-03168-0.

## Introduction

The choice of the best surgical approach in the treatment of temporomandibular joint (TMJ) pathologies is a much-debated topic in literature [1]. To date, the most practiced surgical approach to TMJ is the preauricular one, which provides a wide exposure of all the articular structures [2, 3]. However this approach is complicated by the high risk of neurovascular impairment, salivary fistulae, and facial scarring [3]. The growing attention towards minimally invasive surgery has gradually led to new surgical approaches avoiding aesthetic and functional sequelae typical of TMJ surgeries [4]. The intraoral approach, first reported by Sear in 1972, reduce the risks of facial nerve injury and scarring but offers limited visualization of the operating field [1]. The endoscopic approach gives some advantages as the possibility to perform small incisions, reduced tissue’ damages, and a magnified visualization of the operating field, even in a very narrow space as the temporomandibular joint area [5, 6]. The use of piezoelectric technology has been a great revolution in head and neck surgery due to the simplification of cutting the bone using micro-vibrations [7]. Unlike common bone-cutting tools, piezosurgery offers the benefits of reducing tissue damage, both mechanical and thermal, and can be applied even in very restricted areas [4, 8, 9]. The introduction on the market of piezoelectric handpieces with long tips has opened up new scenarios in the field of minimally invasive surgery [10]. These new tools have allowed the treatment of pathologies in anatomical regions difficult to access like paranasal sinus and skull base diseases, or temporomandibular joints (TMJ) diseases like condylar benign or malign neoplasm, TMJ ankylosis, and condylectomy [3, 6, 9]. To the best of our knowledge this I the first cadaveric study aimed to evaluate and describe the technical feasibility of the intraoral endoscopically assisted condylectomy using the long tip piezoelectric handpiece.

## Case Report

### I - Preoperative Preparations

**Fig. 1:**
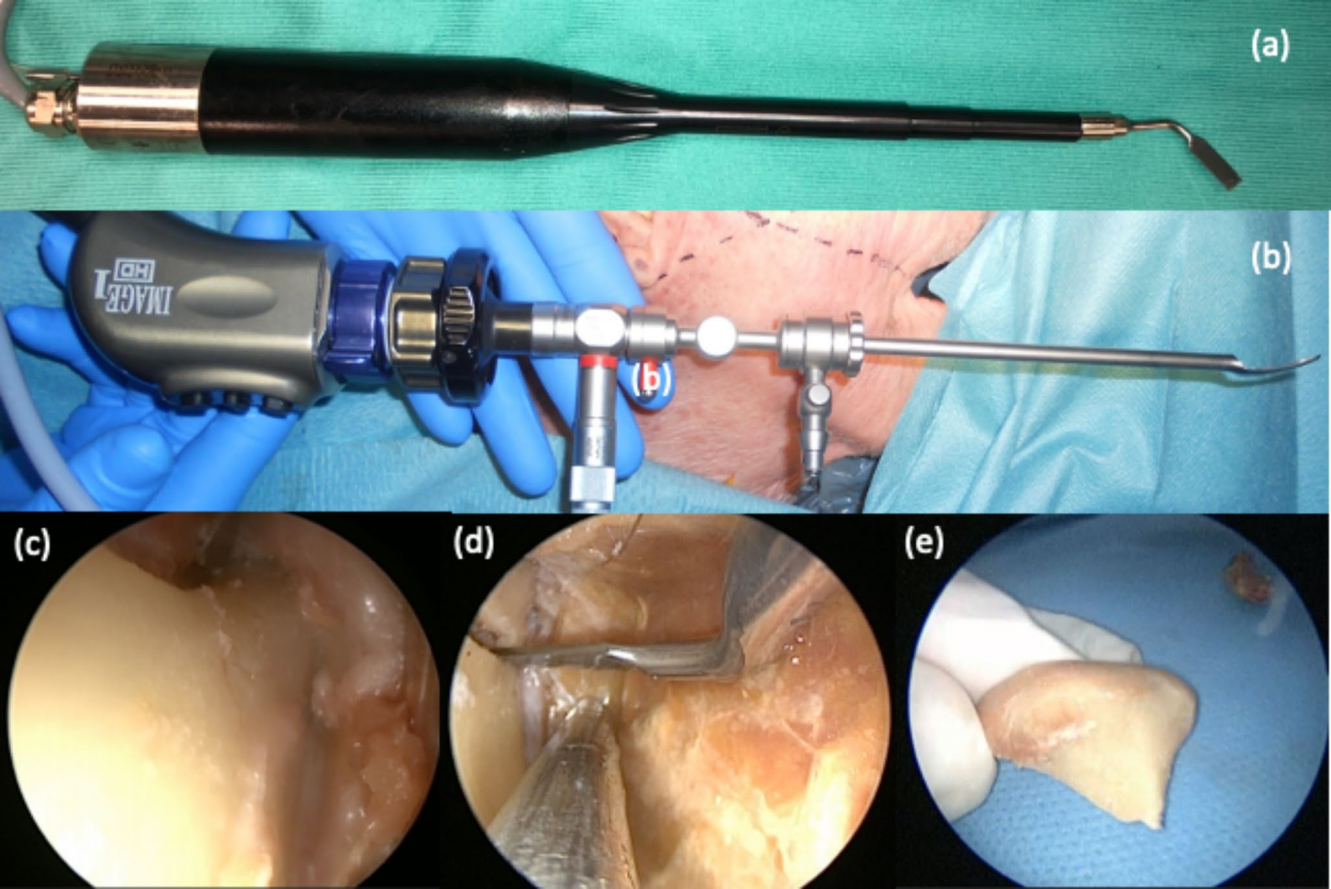
(a) Piezosurgery Plus (Mectron s.p.a. 2014)with a long angled tip (MT5-10 L); (b) HOPKINS rod lens endoscopes with a distal spatula (Karl Storz, Tuttlingen Germany, 50,200 ES,); (c) Blunt dissection of the right mandibular ascending ramus; (d) piezosurgery osteotomy orientation; (e) cut condyle extraction

The procedures have been performed with the aid of 4 mm diameter, 18 cm length rigid, HOPKINS rod lens endoscopes with a 30-degree vision, (Karl Storz, Tuttlingen Germany). An optical dissector (50,200 ES), with a distal spatula, was used to obtain a virtual space for dissection. The dissections were documented by high definition camera and AIDA recording system (Karl Storz®). A piezosurgery (Piezosurgery Plus, Mectron s.p.a. 2014, obtained through a free donation from ANEMA onlus) with a long angled tip (MT5-10 L) was used to perform an intraoral endoscopically assisted low condylectomy. (Fig. 1a)

## II - Surgical Dissection Procedure

**Fig. 2:**
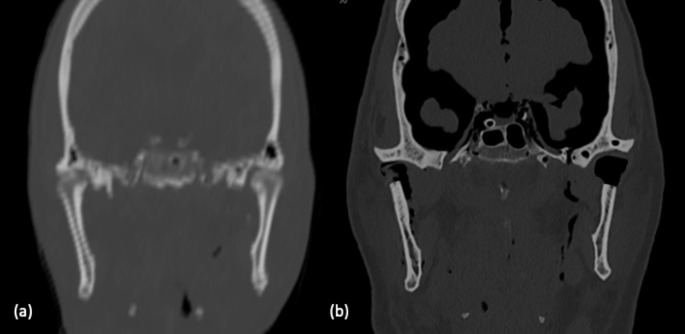
(a) Preoperative CT scan in frontal view; (b) postoperative CT scan in frontal view

With the assistance of endoscopic magnification, a cold blade incision was performed along the external oblique line of the mandible, to expose the anterior margin of the ascending mandibular ramus. The neck of the condyle was reached by blunt tip dissection from the lateral aspect of the ascending branch (Fig. 1c). The piezosurgery (Piezosurgery Plus, Mectron s.p.a. 2014) with a long angled tip (MT5-10 L) was used to perform a low condylectomy (Fig. 1b). The tip of the piezosurgery was oriented perpendicular to the axis of the ramus (Fig. 1d). Through a blunt-tip dissector, the condyle head was freed from temporomandibular ligaments and external pterygoid muscle insertions. The condyle was then extracted with a long tip Klemmer forceps (Fig. 1e). The transoral endoscopic piezosurgery condylectomy was performed bilaterally. Computer tomography scans (CTs) of the head were performed before the surgical treatment and repeated after the procedure (Fig. 2a,b). The access to the TMJ was small according to minimally invasive aim, no significant loss of bone along the osteotomy line was observed. The procedure was completed in about 45 min. The choice of a 30° endoscope with an Optical dissector (50,200 ES), and a distal spatula provided an adequate visualization of the main anatomical structures like the neck of the mandibular condyle, TMJ capsule, insertion of the external pterygoid musculature. The postoperative CT showed a clear osteotomy at the level of both mandibular condyles.

## Discussion

The surgical approaches to the temporomandibular joint are very challenging due to the proximity to the facial nerve [1, 3]. Literature is divided between the external preauricular and the intraoral approaches [1, 2]. The external approaches in all their variants are the most performed but are burdened by a variable incidence rate (from 1 to 32%) of facial nerve injuries and anesthetic scar sequelae due to the cutaneous incision.[2] The first advantages of the intraoral approach were observed by Sear in 1972, followed by Eller in 1977 who used this access to treat a condylar osteochondroma [1]. As reported by Deng et al., the intraoral approach to the TMJ avoids facial nerve impairment (0%) but has a restricted operating field due to the presence of dark corners and undercuts.[1] This limit has been partially exceeded with the introduction of endoscopically assisted surgery [3]. In 2004 Troulis et al. applied the endoscopy to an extraoral submandibular condylectomy, improving the visualization of the operating field thanks to the magnification and ensuring reduced invasiveness of the surgical treatment [6]. Alfaro et al. adopted the intraoral approach in the treatment of condylar hyperplasia (CH) and used the endoscope only in cases where the direct light was not enough for a sufficient view. In these cases (2/7, 28%), a coronoidectomy was necessary to create enough maneuvering space to perform the osteotomy using a reciprocating saw [3]. The introduction of long-tip piezoelectric instruments has opened new frontiers in promoting minimally invasive surgery in the head and neck area [10]. Piezoelectric surgery is used for all the surgeries that interest bone surfaces, and the choice between its different tips represents an advantage where the surgical access is limited and bone is strictly contiguous to soft tissues [9]. Our technical report showed how the introduction of angled endoscopic tools and the use of long tips for piezosurgery effectively compensated the main disadvantages of the TMJ’s intraoral approach. The endoscopic magnification helped to work in a restricted and not well illuminated operating field as the temporomandibular joint. The easy handling and the precision of the long tip piezosurgery allowed to complete the osteotomy procedure in a reduced time and respecting the minimally invasive approach goals. The encouraging results suggest how the described approach can be a valuable alternative to transfacial approaches in the surgical treatment of TMJ pathologies. Further studies are needed to evaluate the application of this technique on a larger court and to other temporomandibular diseases.

## Electronic Supplementary Material

Below is the link to the electronic supplementary material.

A brief video of the procedure is uploaded
